# Placement, support, and retention of health professionals: national, cross-sectional findings from medical and dental community service officers in South Africa

**DOI:** 10.1186/1478-4491-12-14

**Published:** 2014-02-26

**Authors:** Abigail M Hatcher, Michael Onah, Saul Kornik, Julia Peacocke, Stephen Reid

**Affiliations:** 1 Africa Health Placements, North Tower, 3rd floor, 1Sixty Jan Smuts, 160 Jan Smuts Avenue, Rosebank, 2196 Johannesburg, South Africa; 2 Wits Reproductive Health & HIV Institute, Wits Health Consortium, University of the Witwatersrand, 22 Esselen Street, 2001 Hillbrow, Johannesburg, South Africa; 3 Primary Health Care Directorate, Faculty of Health Sciences, University of Cape Town, Observatory, 7925 Cape Town, South Africa

**Keywords:** Community service, Placement, Public sector, Rural, South Africa

## Abstract

**Background:**

In South Africa, community service following medical training serves as a mechanism for equitable distribution of health professionals and their professional development. Community service officers are required to contribute a year towards serving in a public health facility while receiving supervision and remuneration. Although the South African community service programme has been in effect since 1998, little is known about how placement and practical support occur, or how community service may impact future retention of health professionals.

**Methods:**

National, cross-sectional data were collected from community service officers who served during 2009 using a structured self-report questionnaire. A Supervision Satisfaction Scale (SSS) was created by summing scores of five questions rated on a three-point Likert scale (orientation, clinical advising, ongoing mentorship, accessibility of clinic leadership, and handling of community service officers’ concerns). Research endpoints were guided by community service programmatic goals and analysed as dichotomous outcomes. Bivariate and multivariate logistical regressions were conducted using Stata 12.

**Results:**

The sample population comprised 685 doctors and dentists (response rate 44%). Rural placement was more likely among unmarried, male, and black practitioners. Rates of self-reported professional development were high (470 out of 539 responses; 87%). Participants with higher scores on the SSS were more likely to report professional development. Although few participants planned to continue work in rural, underserved communities (n = 171 out of 657 responses, 25%), those serving in a rural facility during the community service year had higher intentions of continuing rural work. Those reporting professional development during the community service year were twice as likely to report intentions to remain in rural, underserved communities.

**Conclusions:**

Despite challenges in equitable distribution of practitioners, participant satisfaction with the compulsory community service programme appears to be high among those who responded to a 2009 questionnaire. These data offer a starting point for designing programmes and policies that better meet the health needs of the South African population through more appropriate human resource management. An emphasis on professional development and supervision is crucial if South Africa is to build practitioner skills, equitably distribute health professionals, and retain the medical workforce in rural, underserved areas.

## Background

Approximately half of the global population lives in rural areas, but these regions have access to less than a quarter of the world’s medical doctors [1]. This geographical and class disparity is most pronounced in resource-constrained settings and has been documented globally [2-6].

South Africa is characterised by inequitable distribution of health services [7], and shifting this trend is a priority for the South African Department of Health (DoH) [8]. Yet while nearly half (approximately 43.7%) of the population lives in rural areas in South Africa, rural communities have access to only 12% of the country’s doctors [8,9]. The majority of South African doctors (70%) work in the private sector, leaving less than 11,000 doctors to serve the 85% of South Africans who do not have private health insurance [10]. There is an average of 13 generalist doctors and 2 specialists available per 100,000 population in rural provinces of South Africa (2008) [11]. The disparities are exacerbated when access to health care is more difficult and costly for rural communities; the impact of transport costs is higher for the rural poor [12]. As a result, the rural population has relatively poor health status. With an inadequate labour force, health care delivery is compromised and poor health indicators impact progress towards the Millennium Development Goals in South Africa.

There is increasing interest in finding effective solutions for attracting and retaining health professionals in rural, underserved areas [13-16]. In response to this perceived need, the World Health Organization has issued recommendations to increase access to health workers in remote and rural areas through improved retention [17-19]. In order to address these gaps in health worker distribution, South Africa is one of 70 countries globally that implement a compulsory community service year [20]. The National Department of Health stated that the main objective of the community service programme is "to ensure improved provision of health services to all the citizens of our country" [21]. This process provides young professionals with an opportunity to develop skills, acquire knowledge, behaviour patterns and critical thinking that will help them in their professional development [21]. Community service for South African health professionals has been implemented since 1998. The programme started with doctors, dentists and pharmacists and, in 2003, grew to include physiotherapists, occupational and speech therapists, clinical psychologists, dieticians, radiographers and environmental health practitioners. A programme for nurses was implemented thereafter [22].

The programme applicants make five choices from a list of public health care facilities approved for community service by the DoH [21]. If allocation is not made within these initial requests, a following set of five choices is available [21]. Participants are legally required to complete a year in community service (remunerative work in the public sector typified by allocated placement) when registering for the first time with their professional council in South Africa [21].

Globally, few programmes define the predicted outcomes of community service or rigorously evaluate the impact of these programmes [20]. The South African community service literature to date has been descriptive in nature [23,24], but comprehensive reviews suggest that retaining health workers in rural, underserved communities requires an understanding of multiple, complex dynamics [15]. We aimed to add to the current evidence base by conducting a national, cross-sectional study among community service participants, specifically doctors and dentists. The community service officer survey was initiated as a mechanism to describe the participants’ perceptions of the programme and its effect in providing access to healthcare for all while enabling the development of these young professionals in South Africa. The survey questions therefore provide an examination of distribution, support and retention of the community service officers. These intentions are corroborated by international evidence that if these programmes are implemented with good planning, transparency and clarity, and support, an impact can be realized in health workforce capacity development, distribution and retention in rural, underserved areas [25]. We anticipated that the results would further inform development of the programme and inform policy makers on implementation of the placement process, development opportunities, and determine retention of community service officers in South Africa.

## Methods

### Research design

We used programme theory assessment [26,27] to drive research questions around community service placement, support, professional development, and retention. The conceptual framework included the sequence of pre-production, production and post-production of human resources. The community service survey aims to evaluate whether we are maintaining quality post-production. According to the 2010 KwaZulu-Natal (KZN) Department of Health [28], the objectives of community service are threefold: a) to ensure equitable distribution of health workers with an emphasis on rural and underserved populations; b) to provide young medical professionals with the opportunity to develop skills and experience to improve their professional development; and c) to enable and encourage community service officers to remain in public service, particularly in rural and underserved areas. In light of these programmatic goals, this report explores three research questions:

a) Distribution: which socio-demographics and medical training characteristics are associated with community service placement in rural areas?

b) Support: which components of community service are associated with professional development?

c) Retention: which factors predict intentions to work in rural, underserved communities?

### Data collection

The data collection tool was a brief, structured questionnaire. Items covered socio-demographics (gender, race, marital status and provincial bursary [29]) and medical training characteristics (profession and medical school attended). A number of items explored characteristics of community service placement, including whether the facility was the participant’s first choice in the allocation process. Rural placement was determined by participants responding that they received a government rural allowance, placement location and level of facility. The DoH provides regulations relating to categories of hospitals where public hospitals are defined as district, regional, tertiary, central and specialized facilities [30]. Additionally, military hospitals/sick bays were included in the survey to accommodate assessment of the South African Military Health Service community service officers. Community health centres/clinics were included in the survey as they form part of hospital clusters approved for the purpose of performing community service.

Placement satisfaction was assessed with a series of items rated on a three-point Likert-type scale (0 = disagree, 1 = neutral, 2 = agree). No participants responded with a neutral answer, therefore all Likert-type items were converted to dichotomous (yes/no) outcomes for analysis. Placement satisfaction included practical items (quality of accommodation, overtime duties, personal safety, fairness of remuneration, and timeous payment of salaries). A Supervision Satisfaction Scale (SSS) was created following data collection to ease interpretation of a number of interrelated supervision factors. Five items were drawn from Saarikoski’s operationalisation of clinical supervision satisfaction [31], which includes ward leadership, aspects of learning in a ward, and the supervisory relationship. The five items adapted for this study included: receiving orientation upon placement, experiencing good clinical supervision, receiving ongoing mentorship, finding clinical leadership accessible, and feeling that concerns were addressed. The SSS was created by summing the scores of the five questions, each of which were rated on a three-point Likert scale.

Future work intentions were assessed by asking participants whether they were considering working in a rural, underserved community in the future (0 = no, 1 = not sure, 2 = yes).

The survey was distributed to all medical and dental community service officers in South Africa in 2009. Individual contact details were supplied by the Health Professions Council of South Africa (HPCSA), and the questionnaires were sent out through the DoH and the relevant provincial coordinator. The distribution of surveys began at the beginning of November 2009 and data collection closed on 1 April 2010. Several different methods (online, e-mail, fax and post) were made available for the submission of surveys. To encourage submissions and maximize the overall response rate, the completion of the survey was included as part of the HPCSA registration process. With the survey, each doctor and dentist received two HPCSA forms (Form 11b - Application for Registration as an Independent Practitioner and Form 27 - Certificate of Completion of Community Service). Upon submission of these forms, the community service officers were also requested to submit their community service research survey responses. In the final stages of the data collection period, all community service officers for whom the team had telephone numbers were called and, if they had not already submitted the questionnaire, they were given the opportunity to complete it telephonically.

### Data analysis

Data were cleaned and entered into Microsoft Access, analysed, and managed in Stata 12, a statistical software package commonly used in health sciences. Descriptive data (frequencies and proportions) were conducted for the entire cohort, and the participating respondents (Microsoft Corporation, Redmond, WA, USA: http://office.microsoft.com/en-za/access/; StataCorp LP, 4905 Lakeway Drive, College Station, Texas 77845–4512 USA: http://www.stata.com/).

Unadjusted logistic regression identified bivariate associations between the outcome of interest and key predictors, with results presented here in the form of an odds ratio (OR). All items with a bivariate association of statistical significance (assessed as *P* < 0.05) were brought into the final multivariate models. Multivariate logistic analyses present adjusted OR (AOR), signifying the statistical association between predictors and outcomes while controlling for all factors.

### Research ethics

The protocol was approved by the University of KwaZulu-Natal Biomedical Research Ethics Committee (Reference BE221/09). The survey was anonymous and the covering letter containing information about the study made it clear that completion of the questionnaire implied consent. To maintain confidentiality, completed questionnaires were sent directly to a researcher at a nongovernmental organization that is independent of the HPCSA, namely Africa Health Placements (AHP). Alternatively, participants could return their questionnaires directly to the HPCSA together with the HPCSA registration forms. After the data were entered into a database, the completed surveys were securely stored at AHP offices until they were transferred to an offsite document storage facility.

### Limitations

Findings from the 2009 community service survey should be viewed in light of several design limitations. The response rate was low, limiting the ability to generalize these findings across the entire community service cohort and may have created a response bias. This is of particular note for dentists. A low response rate is consistent with past community service surveys [23,32], but may be worth exploring further in future studies. Given that the data were programmatic evaluation, and not conducted in a research setting, missing responses were common. This may limit the representativeness of the data presented here. Because structured questionnaires were self-reported, there may be a trend towards positive self-report bias if participants hoped to represent their experience in a more positive light. However, the option to submit the questionnaire to an external stakeholder (AHP) may have ameliorated this limitation. Challenges existed in data collection because of poor communication whereby community service officers or management were unaware of the survey and/or its implications thus affecting the distribution, completion and collection of the survey forms.

Because little demographic data were collected across the entire community service population (n = 1,541), it is challenging to know how the participants (doctors and dentists) differ from community service officers as a whole. However, one important sampling outcome was that participants seemed to respond to the survey whether or not they were placed in their first choice of community service facility. This is important because it suggests that this group may have comprised a representative sample of satisfaction levels.

Several key questions were not asked in the survey. Participants were not asked to report their place of origin/birth (whether in an urban or rural setting), which could assist to explain participant preference for certain locations or their intention to stay in rural, underserved areas in the future [33]. Likewise, language skills were not assessed by the survey, but may lead to important consequences in terms of community service satisfaction. Additional questions about future work intentions in relation to staying in the public sector and remaining in South Africa will be analysed in a separate manuscript.

A final challenge in interpreting the survey findings lies in the poor documentation of the community service programme. Beyond KwaZulu-Natal, few provinces have a dedicated strategy for implementing community service. Even less is known about how community service officers are placed, what the specific programmatic goals are in individual provinces, and how the programme is evaluated over time by the DoH. Although there are limitations in interpreting and drawing conclusions from the data, this research may provide insight useful for improving the distribution and retention of medical practitioners in the future.

## Results

### Response rate and comparison of sample population with all community service officers

Of a total of 1,541 possible respondents, 685 (44%) completed the 2009 survey (Table 1). In Table 1, we compare the characteristics of those community service officers who responded to the survey (Number responded) to the entire sample of community service officers (Total number). In this paper, we analyse only those who responded to the survey (n = 685) who comprise 44% of the total population. A higher proportion of medical doctors (48%) than dentists (27%) responded to the survey. Students with provincial bursary obligations had a response rate of 54%.

**Table 1:** Response rates for the 2009 community service survey

**Characteristics**	**Total number**	**Number responded**	**Response rate**
Response rate	1,541	685	44%
Medical school characteristics			
Profession			
Medical doctor	1,139	544	48%
Dental practitioner	402	110	27%
Profession not indicated		31	
Institution			
University of Cape Town	164	73	45%
University of the Witwatersrand	220	84	38%
University of KwaZulu-Natal	198	101	51%
University of Limpopo	193	55	28%
University of the Free State	108	64	59%
University of Pretoria	270	101	37%
University of the Western Cape	88	64	73%
Stellenbosch University	173	101	58%
Walter Sisulu University	84	34	40%
Other		6	n/a
Provincial bursary obligation (versus not)	262	142	54%
Allocation choice			
First choice	867	368	42%
Second to fifth choice	443	187	42%
Sixth to tenth choice	230	117	51%

### Socio-demographics of the sample population

As shown in Table 2, the majority of participants were female (59%) and single (65%). Those participants stating white as race comprised the majority of the sample (47%) followed by black (23%) and Indian (21%) participants. Participants were spread across nine universities in South Africa with a range of 5% to 15%. A proportion had provincial bursaries or student loans (21% and 35% respectively).

**Table 2:** Descriptive statistics of community service characteristics

**Characteristics**	**Number**	**Percentage**
Socio-demographic characteristics		
Gender (n = 674)		
Male	278	41%
Female	396	59%
Marital status (n = 679)		
Married	232	34%
Single	447	65%
Race (n = 685)		
Black	160	23%
Indian	143	21%
White	319	47%
Coloured	53	8%
Medical school characteristics		
Profession (n = 654)		
Medical doctor	544	79%
Dental practitioner	110	16%
Tertiary institution (n = 677)		
University of Cape Town	73	11%
University of the Witwatersrand	84	12%
University of KwaZulu-Natal	101	15%
University of Limpopo	55	8%
University of the Free State	64	9%
University of Pretoria	101	15%
University of the Western Cape	64	9%
Stellenbosch University	101	15%
Walter Sisulu University	34	5%
Provincial bursary obligation (versus not) (n = 670)	142	21%
Student loans (versus not) (n = 678)	142	35%

### Distribution of health professionals

This section examines how evenly distributed health professionals were across urban and rural sites across South Africa (as defined by the community service officer).

Table 3 illustrates where participants were placed during the community service year. More than half of participants (55%) were placed in rural facilities and 45% were placed in urban facilities. The majority of community service officers were assigned to district or regional hospitals (39% and 26% respectively). Eighteen percent of community service officers were assigned to central or tertiary hospitals, four percent to military hospitals and three percent to specialized hospitals. Ten percent of community service officers were allocated to community health centres or clinics.

**Table 3:** Descriptive statistics of community service training

**Characteristics**	**Number**	**Percentage**
Community service training characteristics		
Placement (n = 677)		
Urban	305	45%
Rural	372	55%
Level of facility (n = 672)		
Specialized hospital	12	3%
Central/Tertiary hospital	72	18%
Regional hospital	104	26%
District hospital	156	39%
Military hospital/Sick bay	16	4%
Community health centre/Clinic	40	10%
Province (n = 682)		
Eastern Cape	72	18%
Free State	12	3%
Gauteng	44	11%
KwaZulu-Natal	96	24%
Mpumalanga	28	7%
Northern Cape	40	10%
Limpopo	20	5%
North West	8	2%
Western Cape	81	20%
Allocation choice (n = 672)		
First choice	368	55%
Second to fifth choice	187	28%
Sixth to ninth choice	71	11%
Tenth choice	46	7%

The majority of participants (55%) were assigned to their first choice of placement, after which 28% were placed within their top two to five choices.

**Figure 1:**
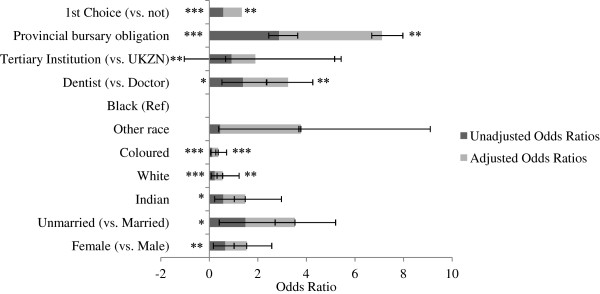
**Unadjusted (dark grey) and adjusted (grey) associations between rural placement and key socio-demographic and medical school characteristics as predictors (n = 639).** Data are presented as odds ratios. Ninety-five percent confidence intervals are illustrated by error bars. UKZN: University of KwaZulu-Natal. Statistical significance is presented with an asterisk to indicate the relative level of statistical significance (*P < 0.05 (statistically significant), **P < 0.01, ***P < 0.001 (statistically highly significant).

In an effort to understand how community service officers are placed in rural or urban environments and what factors predict these associations, we examined the association between rural placement and various socio-demographics and medical school characteristics (Figure 1). In unadjusted models, females were less likely than males to be placed in rural facilities (OR 0.65, 95% CI 0.48 to 0.89). Unmarried community service officers were more likely than their married counterparts to be placed in a rural facility (OR 1.49, 95% CI 1.08 to 2.04). Race^a^ was a significant predictor of where community service participants were placed. Compared to community service participants stating black as race, white (OR 0.22), Indian (OR 0.57), and coloured (OR 0.12) participants were disproportionately less likely to be placed in a rural setting; however a response bias may influence this finding. It was not possible to determine the extent to which this racial variability was linked to home language or place of birth.

Adjusted models show that those participants stating white or coloured as race were disproportionately less likely to receive a rural placement (AOR 0.39, 95% CI 0.23 to 0.68 and 0.15, 95% CI 0.10 to 0.34 respectively) (Figure 1), even when controlling for other socio-demographics, medical school characteristics, and choice of facilities. Dentists had greater odds of receiving a rural placement (AOR 1.67, 95% CI 1.19 to 2.35), even when considering other key participant traits. Those with provincial bursaries were twice as likely to receive a rural placement compared to those without bursaries (AOR 2.08, 95% CI 1.23 to 3.53). Even when controlling for other characteristics, those who received their first choice of placement were significantly less likely to ‘go rural’ (AOR 0.60, 95% CI 0.42 to 0.86).

**Figure 2:**
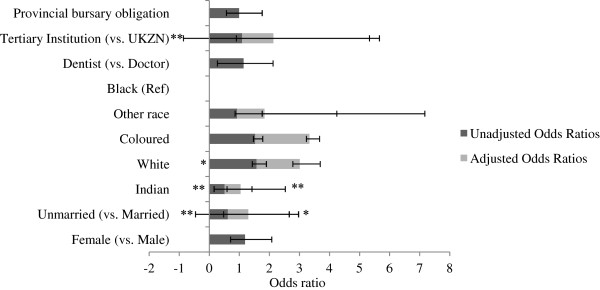
**Unadjusted (dark grey) and adjusted (grey) associations between receiving first choice of placement and key predictors (n = 670).** Data are presented as odds ratios. Ninety-five percent confidence intervals are illustrated by error bars. UKZN: University of KwaZulu-Natal. Statistical significance is presented with an asterisk to indicate the relative level of statistical significance (*P < 0.05 (statistically significant), **P < 0.01, ***P < 0.001 (statistically highly significant).

Unadjusted associations show that although receiving a first choice of placement did not differ across gender (OR 1.19, 95% CI 0.87 to 1.62), it was significantly different across marital status (OR 0.62, 95% CI 0.46 to 0.86) (Figure 2). Unmarried community service officers were less likely to receive their first choice compared to married officers. In terms of racial demographics, Indians were half as likely as black counterparts to receive their first choice of placement (OR 0.51, 95% CI 0.32 to 0.82), whereas whites were more likely (OR 1.57, 95% CI 1.06 to 2.31).

Adjusted analysis suggests that being unmarried reduced one’s odds of receiving a first choice in placement by 32% (OR 0.68, 95% CI 0.49 to 0.95). Even when controlling for other factors, Indians were disproportionately unlikely to receive their first choice (OR 0.53, 95% CI 0.32 to 0.85). However, the other predictors did not significantly predict first choice placement.

### Satisfaction of participants with their community service training and mentorship

Overall, participants reported high levels of satisfaction with community service training and mentorship. Table 4 describes overall satisfaction with supervision, management, and practical concerns during the community service year. A large majority of participants reported that they had experienced professional development and reported making a community contribution during the year (87% and 95% respectively).

**Table 4:** Descriptive statistics of community service satisfaction and future work intentions

**Characteristics**	**Number**	**Percentage**
Experienced professional development (n = 539)	470	87%
Contributed to health of community (n = 595)	563	95%
Supervision satisfaction		
Well-oriented to job (n = 537)	465	87%
Good clinical supervision (n = 494)	350	71%
Good mentorship and support (n = 459)	329	72%
Seniors available (n = 513)	420	82%
Management handled concerns well (n = 416)	209	50%
Practical satisfaction		
Performed overtime duties (n = 632)	570	90%
Satisfied with accommodation (n = 408)	233	57%
No risk to personal safety (n = 457)	236	34%
Remuneration is fair (n = 448)	242	54%
Received salary on time (n = 605)	465	77%
Where planning to work next year		
Private sector, SA (n = 657)	150	23%
Public sector, SA (n = 657)	453	69%
Overseas (n = 657)	39	6%
Other (n = 657)	15	2%
Intention to specialize (n = 620)	419	68%
Intention to stay at same CS facility (n = 620)	203	34%
Intention to work in rural or underserved communities	171	25%

CS: community service.

In terms of supervision and management, most felt well-oriented to the job (87%) and reported that seniors were available when needed (82%). Approximately three quarters felt they had good clinical supervision (71%) as well as satisfactory mentorship and support (72%). However, only half felt that management handled concerns well.

Most participants reported working overtime (90%) with the majority receiving their salaries on time (77%). However, approximately half reported accommodation as unsatisfactory (43%), their personal safety as lacking (66%), and remuneration as unfair (46%). A small majority (66%) would recommend their community service facility to others, but only one-third intended to stay at the same facility in the coming year (34%).

Supervision satisfaction was reported as worse in rural areas in bivariate analysis (OR 0.43, 95% CI 0.29 to 0.65) (n = 677). In adjusted analysis, the association persisted, even when controlling for basic demographics (gender, marital status, profession and tertiary institution) (AOR 0.41, 95% CI 0.27 to 0.62).

### Factors that contributed to satisfaction in community service professional development

**Figure 3:**
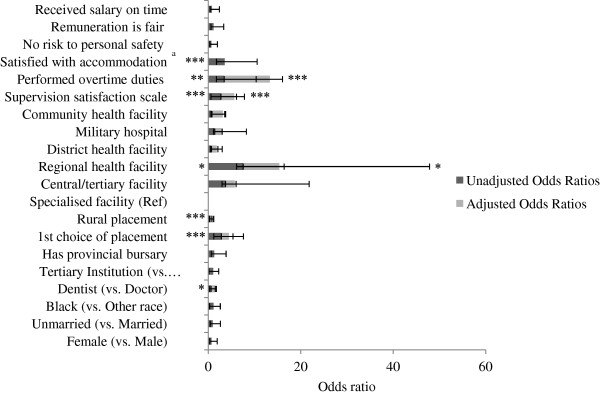
**Unadjusted (dark grey) and adjusted (grey) associations between professional development and key predictors (n = 483).** Data are presented as odds ratios. Ninety-five percent confidence intervals are illustrated by error bars. UKZN: University of KwaZulu-Natal. ^a^Variable omitted from multivariate model due to colinearity with level of health facility. Statistical significance is presented with an asterisk to indicate the relative level of statistical significance (*P < 0.05 (statistically significant), **P < 0.01, ***P < 0.001 (statistically highly significant).

In Figure 3, we explored how socio-demographics, placement characteristics, and management experiences were associated with community service officer satisfaction. The outcome was a dichotomous measure of whether participants reported that they ‘experienced significant professional development’ during the year.

In unadjusted models, socio-demographics did not seem to play a role in how community service officers reported their professional development experience. However, dentists were less likely than doctors to report adequate professional development (OR 0.59, 95% CI 0.39 to 0.90). Those in regional health facilities were almost eight times more likely to report significant professional development than those in specialized facilities^b^ (OR 7.52, 95% CI 1.41 to 40.33). Rural placement was negatively associated with professional development (OR 0.29, 95% CI 0.16 to 0.47), however when controlling for other variables this association was attenuated. In comparison with the Eastern Cape, participants placed in Mpumalanga and KwaZulu-Natal experienced worse professional development (OR 0.13, 95% CI 0.04 to 0.40; OR 0.37, 95% CI 0.13 to 1.01 respectively) (data not shown). The SSS was highly correlated with participant professional development in bivariate analyses. Those who rated their facilities better in terms of job orientation, clinical supervision, mentorship, and management were more likely to indicate that they experienced professional development during the community service year (OR 2.69, 95% CI 2.14 to 3.39). Those who worked overtime and those who were satisfied with accommodation were more likely to report development (OR 3.41, 95% CI 1.68 to 6.93 and 3.54, 95% CI 1.79 to 7.00 respectively).

Adjusted models show that even when controlling for other important factors, regional health facilities strongly predicted a report of good professional development (AOR 7.83, 95% CI 1.04 to 58.64) when compared to specialized facilities. SSS retained its significance in adjusted models, with each step upwards on the scale being associated with three times higher odds of reporting professional development (AOR 2.93, 95% CI 2.18 to 3.95). Lastly, those reporting they had worked overtime were nearly ten times as likely to report professional development (OR 9.92, 95% CI 2.71 to 36.38).

### Retention of health professionals

**Figure 4:**
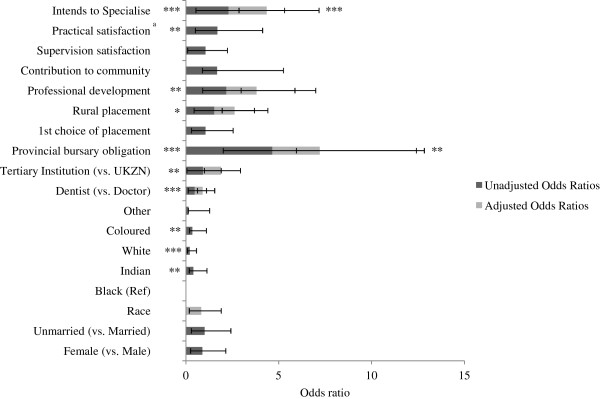
**Unadjusted (dark grey) and adjusted (grey) associations between public sector work intentions and key predictors (n = 469).** Data are presented as odds ratios. Ninety-five percent confidence intervals are illustrated by error bars. UKZN: University of KwaZulu-Natal. ^a^Variable omitted from multivariate model due to missing data. Statistical significance is presented with an asterisk to indicate the relative level of statistical significance (*P < 0.05 (statistically significant), **P < 0.01, ***P < 0.001 (statistically highly significant).

This section explores future work intentions among community service participants (working in a rural or underserved community or working in the public sector). The majority of participants planned to work in South Africa following the community service year, with most (69%) indicating they plan to work in the public sector and one fifth (23%) opting for work in the private sector (Table 4). However dentists were half as likely to work in the public sector compared to doctors (Figure 4). The majority of participants intended to specialize (68%). One quarter of participants intended to work in rural or underserved communities. Unlike previous surveys [32], very few participants (6%) intended to work overseas during the coming year. Another divergence from existing data is the high proportion (34%) of community service officers intending to remain at the same facility [23]. It was noted that 29.6% of those with provincial bursary obligations indicated intentions to work in the public sector the following year, even though public service is a compulsory requirement.

Unadjusted analysis shows a number of factors predict intentions to work in the public sector (Figure 4). Black participants were more likely than their counterparts to report intentions of working in the public sector. University of KwaZulu-Natal medical school graduates were more likely than graduates from other universities to plan on public sector work. Gender and marital status did not seem to correlate with intentions to work in the public sector (OR 0.89, 95% CI 0.64 to 1.26, OR 1.01, 95% CI 0.72 to 1.41 respectively).

Participants who were placed in rural facilities were significantly more likely to plan for public sector work in the future (OR 1.53, 95% CI 1.09 to 2.16), as were those with provincial bursary obligations (OR 4.65, 95% CI 2.64 to 8.19). Those intending to specialize were more likely to have intentions of working in the public sector (OR 2.30, 95% CI 1.76 to 3.01), as were those who reported higher levels of professional development (OR 2.18, 95% CI 1.28 to 3.69) and practical satisfaction (OR 1.70, 95% CI 1.19 to 2.43). It is important to consider that specialization is undertaken at tertiary facilities **-** all of which are part of the public sector in South Africa.

Adjusted analysis shows that being a dentist made participants half as likely to seek public sector work (AOR 0.43, 95% CI 0.28 to 0.65). Having a provincial bursary more than doubled the odds of public sector work (AOR 2.56, 95% CI 1.26 to 5.20) as did intention to specialize (AOR 2.05, 95% CI 1.49 to 2.82). The relationship between all other potential factors (race, tertiary institution, rural placement and professional development) was attenuated once adjusting for other predictors.

Intention to remain in the same facility was associated with supervision satisfaction (OR 1.25, 95% CI 1.14 to 1.39) and allocation choice (OR 1.60, 95% CI 1.13 to 2.28) (data not shown). However in an adjusted analysis accounting for socio-demographics supervision satisfaction alone predicted intention to remain in the same facility (AOR 1.23, 95% CI 1.10 to 1.37).

**Figure 5:**
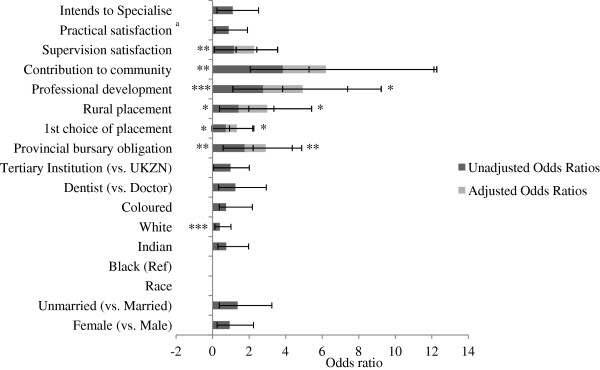
**Unadjusted (dark grey) and adjusted (grey) associations between intention to work in rural or underserved communities in the future and key predictors (n = 460).** Data are presented as odds ratios. Ninety-five percent confidence intervals are illustrated by error bars. UKZN: University of KwaZulu-Natal. Statistical significance is presented with an asterisk to indicate the relative level of statistical significance (*P < 0.05 (statistically significant), **P < 0.01, ***P < 0.001 (statistically highly significant).

Our finding that only one quarter of participants (25%) intended to work in rural or underserved communities in the future is consistent with past community service surveys (Table 4), [32,34]. Intentions to work in rural or underserved areas in the future (Figure 5) seem to be associated with a number of factors. Unadjusted analysis shows that participants stating white as race were less likely than their counterparts to plan for rural or underserved work (OR 0.40, 95% CI 0.27 to 0.61). It was observed that 65% of participants with provincial bursary obligations were placed in a rural location and were more likely to intend to work in a rural location in the future (OR 1.75, 95% CI 1.17 to 2.61). In adjusted analysis the association with intention to work in rural or underserved areas is attenuated when controlling for race (data not shown) as black participants were more likely to receive provincial bursaries and intend to ‘go rural’ (Figure 5).

If community service officers were placed in a rural facility during their community service year, they appear more likely to plan for work in rural or underserved communities in the future (OR 1.42, 95% CI 1.04 to 1.94), as were those who were satisfied with community service supervision (OR 1.16, 95% CI 1.06 to 1.26) and those who reported significant professional development (OR 2.76, 95% CI 1.65 to 4.63). Those receiving their first choice in community service placement were less likely to plan to work in rural or underserved communities in the future (OR 0.72, 95% CI 0.76 to 1.48).

In adjusted analysis, those receiving their first choice of placement in the community service year were 39% less likely to plan to work in rural or underserved communities (AOR 0.61,95% CI 0.40 to 0.93), even when controlling for other factors. Rural placement during the community service year increased the odds of work intentions in rural or underserved communities (AOR 1.57, 95% CI 1.01 to 2.43). Adjusted analysis suggests that race, provincial bursary obligations, contribution to community, and supervision satisfaction were no longer significant predictors of rural or underserved community work intentions. When controlling for placement in an urban or rural setting, community service officers were more likely to intend to work in rural or underserved areas if they experienced good professional development (AOR 2.17, 95% CI 1.10 to 4.30).

## Discussion

### Refining a community service placement strategy and guiding future work intentions

This study of community service officers is among the first to use multivariate statistical methods to parse out the influence of multiple factors on the placement, professional development, and retention of health professionals in South Africa.

### Placement

Placement data suggest a strategy to increase the health workforce in the rural environment. However, while district level facilities encompass small, medium and large hospitals reflecting allocation to some rural locations, it remains apparent that there is a large proportion of community service officers allocated to urban facilities. Rural placement by participant choice was viewed as undesirable within this sample.

The intention of the provincial distribution may be according to the human resource needs of the target populations. According to a needs and gaps analysis published in 2009 [10] there was a widespread decline in density ratios of medical practitioners in all provinces. The Eastern Cape, Limpopo, Mpumalanga and the Northern Cape had the lowest number of medical practitioners per 100,000 people, which is expected as these provinces are more rural [10]. These provinces, together with KwaZulu-Natal, had the greatest number of medical practitioner vacancies in 2008 [10]. The distribution of community service officers suggests an attempt to meet the needs of each province. However, the adequacy of such a distribution is a subject of future research.

### Support

A 50% participant response that management handled concerns well suggests this could be a potential area of focus to improve participant satisfaction. The data on supervision satisfaction suggest that accommodation, personal safety and remuneration could be potential areas of focus to increase incentive for community service.

The proportion of participants reporting professional development (87%) is higher than earlier surveys (compared with 64% and 71%, in 1999 and 2001 respectively) [32]. Professional development reports were observed to be predicted by regional health facilities. Health facilities differ in the complement of equipment or resources and the presence of a satisfactory staff (that is a ratio of cadres enabling optimal support and professional development) and thus some facilities offer better supervision than other facilities. These factors may be optimal in a regional health facility. However, the findings suggest that all facilities, regardless of size, are able to encourage professional development by meeting five key areas of the SSS: orientation, clinical advising, mentorship and support, accessibility by senior leadership, and handling concerns. The fact that supervision satisfaction predicted professional development is hardly unexpected, given the evidence that institutional support and active supervision seem to be key components of a positive community service experience [24,35]. However, to our knowledge this is the first analysis that has combined these five items into a codified scale. The SSS could be useful for other programmes aiming to measure the clinical supervision and mentorship of junior practitioners.

Interestingly, most literature to date assumes that high workloads are either a burden on community service officers [36], or a gross violation of human rights and labour laws [37]. Yet, our findings suggest overtime work is associated with better development. This comparison is limited because the amount of ‘overtime work’ can be highly variable and must be clearly defined as a specific number of hours in the community service survey. While certain levels of overtime can be beneficial for professional development, extensive hours may have a negative effect on professional development and therefore further research is required to further describe this association.

Our finding that the majority of participants (95%) felt they had made a difference in the community they served is consistent with other studies in South Africa. Among psychologists, 90% felt they made a contribution to the community, notwithstanding difficulties around accessing materials, finding accommodation, and clarifying their role during the community service year [38]. Drivers of community contribution were: having a provincial bursary, supervision satisfaction, and performing overtime duties. This suggests that active facilities staffed by well-supervised practitioners will most likely provide a feeling of community contribution during the community service year. It is important to note that the corollary of community contribution has been identified in studies within the receiving communities, who feel that community service officers ‘make a difference’ [39].

### Retention

Key drivers of rural or underserved community work intentions emerged in this survey, and can be capitalized upon in future community service strategies. For one, reporting significant levels of professional development doubled the odds that participants planned to work in rural or underserved communities even when controlling for key demographics. Capitalizing on professional development during the community service year could have a significant impact on the practitioners who are retained in rural or underserved areas in the future. The Health Professions Council of South Africa (HPCSA) conducts Continuing Professional Development (CPD) learning activities [40]. Community service health professionals are not required to comply with CPD requirement but are encouraged to attend [40]. The impact of such programmes on the professional development experience of community service officers requires further evaluation. Poor management, assessed as a factor of the SSS which predicted professional development, provides an area for improvement that may affect retention in rural or underserved areas.

Likewise, receiving a rural placement during the community service year improved the odds of a community service officer considering future rural or underserved community work whether or not that was their first choice of placement. This suggests that the community service allocation process in each province should consider placements more strategically in terms of exposing new professionals to rural facilities even if this was not the top choice of placements. In other words, community service officers who remain in their ‘comfort zone’ (as indicated by being assigned to their first choice facility) possibly fail to consider other options for future work. Utilizing the community service year as an opportunity to expose health professionals to rural or underserved community work, while supporting them in professional development, should be a priority for future community service programming.

There is no evidence from these findings that certain types of community service placements ‘immunize’ doctors against future work in the public sector, as has been suggested in literature [41]. Indeed, basic levels of job satisfaction (both in terms of professional development and practical concerns such as accommodation) predicted future work in the public sector among our sample population. However, rural placement and/or a low choice of placement (that is, second through tenth choice as compared to first choice) did not seem to influence public sector intentions in multivariate models. This suggests that the placement itself is less important than the support and satisfaction that participants derive from the community service year in predicting public service. Further investigation will establish whether those who are intending to work in the public sector do so for the purposes of specialization and bursary obligations and their intentions to remain in the public sector thereafter.

## Conclusions

Although the community service programme articulates the goal of ‘equitably’ distributing health professionals throughout the country, our analysis suggests that important inequities deserve attention. If these gender, marital status and racial disparities are intentional (for example, based on language skills or place of birth/origin) then this needs to be made transparent. The data suggest a need to reduce the intentional or unintentional biases that emerge through the placement process. Indeed, transparency of placement processes is noted to be a key ingredient to the success of compulsory community service [20]. Secondly, as others have noted [24], the programme management should better describe the overall programme goal to provide services to underserved communities through more appropriate allocations.

Creating sustainable strategies for attracting and retaining health professionals in rural, underserved communities is no simple fix. Literature urges governments to implement many interventions with regards to living environments, working conditions and professional development opportunities [15,32]. In South Africa, a comprehensive strategy that extends beyond community service, prioritizes access to health professionals in rural and underserved areas [8]. Our findings suggest that South Africa’s community service programme is an excellent recruitment strategy for health professionals. However the programme needs to be complemented by transparent processes that clearly articulate a strategy for placement of community service officers. The programme should facilitate numerous mechanisms for professional development, and employ innovative strategies to improve retention with rigorous identification, implementation and evaluation mechanisms. Such improvements will ensure progress towards optimising the programme and improving access to healthcare in the public service.

## Endnotes

^a^Race was observed in this study as an attempt to define the role of socio-demographics in the distribution of community service officers. The terms were approved as part of the study by the aforementioned ethics committee.

^b^Specialized facilities were used as a reference out of ease because these facilities appeared theoretically different from other types of facilities, but the reference does not affect estimates or direction of association.

## Abbreviations

AHP: Africa Health Placements; CI: confidence interval; CS: community service; DoH: Department of Health; HPCSA: Health Professions Council of South Africa; KZN: KwaZulu-Natal; OR: odds ratio; SSS: Supervision Satisfaction Scale.

## Competing interests

Africa Health Placements works with the Department of Health and the Health Professions Council of South Africa (HPCSA) to plan, find and keep the workforce needed to deliver quality healthcare to South Africa’s rural population. The authors declare that they have no competing interests.

## Authors’ contributions

AMH conceptualised the research approach, led the data analysis and interpretation, and drafted the manuscript. MO advised on data interpretation and performed the statistical analysis. SK conceived the study idea, design of the survey, analysis and interpretation of data, and provided critical revision of the draft manuscript. JP assisted with data interpretation and drafted the manuscript. SR was involved in the design of the questionnaire, determining the areas of research the study needed to address, ethics approval, dissemination of the survey and review of the results. All authors read and approved the final manuscript.
